# Simulation analysis of visual perception model based on pulse coupled neural network

**DOI:** 10.1038/s41598-023-39376-z

**Published:** 2023-07-28

**Authors:** Mingdong Li

**Affiliations:** School of Information Engineering, Suzhou University, Suzhou, 234000 China

**Keywords:** Computer science, Information technology

## Abstract

Pulse-coupled neural networks perform well in many fields such as information retrieval, depth estimation and object detection. Based on pulse coupled neural network (PCNN) theory, this paper constructs a visual perception model framework and builds a real image reproduction platform. The model firstly analyzes the structure and generalization ability of neural network multi-class classifier, uses the minimax criterion of feature space as the splitting criterion of visual perception decision node, which solves the generalization problem of neural network learning algorithm. In the simulation process, the initial threshold is optimized by the two-dimensional maximum inter-class variance method, and in order to improve the real-time performance of the algorithm, the fast recurrence formula of neural network is derived and given. The PCNN image segmentation method based on genetic algorithm is analyzed. The genetic algorithm improves the loop termination condition and the adaptive setting of model parameters of PCNN image segmentation algorithm, but the PCNN image segmentation algorithm still has the problem of complexity. In order to solve this problem, this paper proposed an IGA-PCNN image segmentation method combining the improved algorithm and PCNN model. Firstly, it used the improved immune genetic algorithm to adaptively obtain the optimal threshold, and then replaced the dynamic threshold in PCNN model with the optimal threshold, and finally used the pulse coupling characteristics of PCNN model to complete the image segmentation. From the coupling characteristics of PCNN, junction close space of image and gray level characteristics, it determined the local gray mean square error of image connection strength coefficient. The feature extraction and object segmentation properties of PCNN come from the spike frequency of neurons, and the number of neurons in PCNN is equal to the number of pixels in the input image. In addition, the spatial and gray value differences of pixels should be considered comprehensively to determine their connection matrix. Digital experiments show that the multi-scale multi-task pulse coupled neural network model can shorten the total training time by 17 h, improve the comprehensive accuracy of the task test data set by 1.04%, and shorten the detection time of each image by 4.8 s compared with the series network model of multiple single tasks. Compared with the traditional PCNN algorithm, it has the advantages of fast visual perception and clear target contour segmentation, and effectively improves the anti-interference performance of the model.

## Introduction

In recent years, with the acceleration of information process and the rapid development of computer technology, people's demand for computer vision in life and production is becoming more and more urgent^1^. Computer vision is using computer simulated biological vision system for environmental awareness and understanding, and visual perception as a first step in computer vision system for image processing, computer vision is one of the core technology items^2^. Image completion can often reproduce the lost information^3^, and image completion can fill the defects of the picture when the target features are seriously missing^4^. The problem of image resolution reconstruction can be improved by using deep neural network to process feature data^5^. Real image reproduction is a processing technology to enable imaging equipment to provide ideal images in line with human physiological visual perception^6^.

At present, people have carried out a lot of research work on the difficulties of visual perception technology, and made many breakthroughs. How to process the image information by deep residual group and draw the image by deep learning model, and then repair the image^7,8^, and successfully tested in the application^9–11^. One of the problems is video structure, that is, how to automatically realize video frequency time-domain segmentation, and segment the video stream into different levels of video units with certain significance. The second is how to realize automatic content analysis and extract visual and semantic features to describe video content^12^. But the current evaluation of visual perception algorithm is also a lack of a system, accurate research, how to judge the effect of visual perception and how to choose appropriate for certain types of image segmentation algorithm is also does not have a unified standard^13^, so research and design segmentation algorithm performance evaluation criteria system, the current field of visual perception is an urgent need to solve the problem^14^.

Based on the theory of pulse coupled neural network, this paper constructs a visual perception model, and analyzes the performance evaluation criteria of existing visual perception algorithms in detail. This paper discusses four calculation methods of visual boundary coefficient, and proposes two detection methods of visual boundary based on the model^15^. Because the method considers multiple frames at the same time, it has good anti-noise ability, and the recall and accuracy of visual boundary detection are better than the traditional visual boundary detection method based on the difference of adjacent frames. During the experiment, the source image is decomposed into high and low frequencies in the non-downsampled shear-wave transform domain. Then, the improved pulse coupling neural network is used to sense the low frequency subband coefficients, and the sum of the squared variance of pixels is used as its excitation, and the sum of the direction gradient is selected as its connection strength. The high frequency subband coefficients with large calculation amount are processed by compressed sensing^16^. Finally, the perception image is obtained by non-subsampled shear wave inverse transform, and the optimal value range of parameters is obtained by relevant experiments^.^

In this paper, the main contributions are: (1) This research starts from the stereo matching problem itself and establishes an appropriate mathematical model to solve it. Stereo vision is the description function of human vision, the basic function of human visual system. The human visual system can achieve stereo matching quickly and accurately. Although there are still some shortcomings in artificial systems to simulate human vision, it is promising to explore vision algorithms from the perspective of vision. Pulse coupled neural network (PCNN) model is transformed from visual cortex model and has specific ability of image feature processing. (2) The stereo matching algorithm combines PCNN and Markov random field, and uses the likelihood probability model based on the similarity evaluation of PCNN. The two images are sent to two PCNNS respectively, and the pulse sequence is generated after several iterations. Then BP algorithm is used to achieve the maximum a posteriori probability, and good matching results are obtained. (3) According to the coupling characteristics of PCNN, the tightly connected space and gray level characteristics of the image, the local gray mean square error of the connection strength coefficient of the image is determined to achieve fast visual perception and clear object contour segmentation. After research, PCNN visual perception inference model of stereo matching algorithm can be very good enough to apply to the real object detection, has a very wide application prospect.

## Related work

At present, the theoretical and application research on the performance evaluation criteria of visual perception algorithms mainly starts from the following two aspects: one is to judge the comprehensive performance of a segmentation algorithm for different kinds of visual perception, and on this basis, the parameters and models of the algorithm are improved to expand its application scope^17^. Second, different types of segmentation algorithms are selected to cut the same image, and the performance of each segmentation algorithm is judged by analyzing the segmentation results to determine the optimal segmentation algorithm^18^.

Su^19^ proposed to use the level set function to indirectly express the model contour, so as to indirectly realize the purpose of updating the contour by updating the level set function. Therefore, when the topology structure of the contour changes, the level set function can still maintain validity, and successfully solve the problem of the topology change of the contour. At the same time, compared with the parametric active contour model, it can realize the simultaneous segmentation of multiple complex targets. Jing^20^ proposed the elaborate design of three dimension of depth pulse coupling network cascade structure, using the step by step increase the input to the scale from coarse to fine in predicting the location of human face and pedestrians, the statistics in the picture all the number, and accurate human face image with more original scale application in human face detection, recognition of gender and age recognition key multitasking recognition system^21^. Using multi-task and multi-scale method to train the network, the detection accuracy of face detection, pedestrian detection, face key point detection, gender and age detection has been improved to a certain extent, and the detection speed has been greatly improved.

Panigrahy^22^ proposed a classical model based on region, commonly known as the C-V model. Different from the parameter and level set active contour model, the C-V model does not rely on the gradient information of the image for visual perception. Yang^23^ believed that the C-V model could also obtain good segmentation results for images with meaningless gradients and fuzzy boundaries, and it successfully solved the problem of boundary dependent segmentation of level set active contour model based on boundaries. Since the C-V model is evolved on the basis of the Mumford-Shah model^24^, the Mumford-Shah model is briefly introduced before the introduction of the C-V model. Researchers proposed a superpixel method to maintain color homogeneity based on global and local boundary advancement of watershed transformation^25^. In the first stage, the flooding priority is calculated by spreading from seed to other pixels. In the second stage, boundary pixels are defined by two separate criteria, one focusing on color uniformity and the other on shape regularity, flooding from the initial boundary pixels toward pixels that are more likely to be true. The final segmentation result maintains color uniformity in the content rich region, and improves the regularity of superpixels in the content flat region^26^. Pulse coupled neural network (PCNN) is often used to generate fused images by fusion rules, but its performance is sometimes controlled by the selection of parameters. Recently, Yin applied PCNN with adaptive parameters to image fusion and achieved good results. Estimation methods based on differential box counting are widely accepted for quantifying the texture of an image. In the WPADPCNN model, the adaptive estimation parameters are found from the input, and the FD weighting is calculated. Experiments show that MRI and SPECT images perform better in visual quality and image clarity than the experimental results.

## Visual perception model construction based on pulse coupled neural network

### Neural network dynamics

The channels for each pulse element to receive external stimulus input in PCNN include feedback input channels and connection input channels. Moreover, the internal active item U of the pulse element is modulated by the nonlinear multiplication of the inverse feed input item F and the connection input item. U stands for nonlinear modulation matrix.Whether the pulse is issued in PCNN is related to the internal activity item U and threshold E of the neuron. Each pulse coupling kernel has a size, and the size of the six pulse coupling kernels in layer C1 is 5 × 5. The function f represents the pixel value of the coupled pulse image.The pulse coupling kernel is used to slide on the input data f(i, j) according to a fixed step size u(i) to make the pulse coupling kernel calculate the pulse coupling on the local data f(i).

1 $$ \frac{1}{1 - n}\sum {\frac{f(i,j) - u(i)}{{f(m) - f(n)}} < n} $$

2 $$ 1 - |x| > \frac{1}{1 - n}\ln |x - f(j - 1)| $$

In the process of sparse decomposition 1-|x|, the high-frequency coefficient of multi-scale decomposition represents the detailed information such as region boundary and edge of multi-source image, and the human visual system is sensitive to the detailed information such as edge. How to construct high frequency coefficient perception strategy and extract significant high frequency coefficient is very important to improve the quality of perception image. Combined with the characteristics of high frequency component of source image w(s, t), image quality evaluation factor p(x, y) is considered to construct perception strategy.

3 $$ w(s,t) - w(s,0) = w(s - 1,t - 1) $$

4 $$ \sum {p(x,y) - p} (x - x^{2} ) < p[n - 1] $$

In PCNN network, each pixel in the image is equivalent to an impulse element. At this point, the threshold E increases rapidly through the feedback input, causing the pulse element to stop transmitting pulses. The threshold k(x)/k(y) begins to decay over time, and when it is again smaller than the internal active term, the pulse element fires again, and so on.

5 $$ \sum {k(x)} /k(y) < \log (x - x^{2} - y - 1) $$

**Figure 1:**
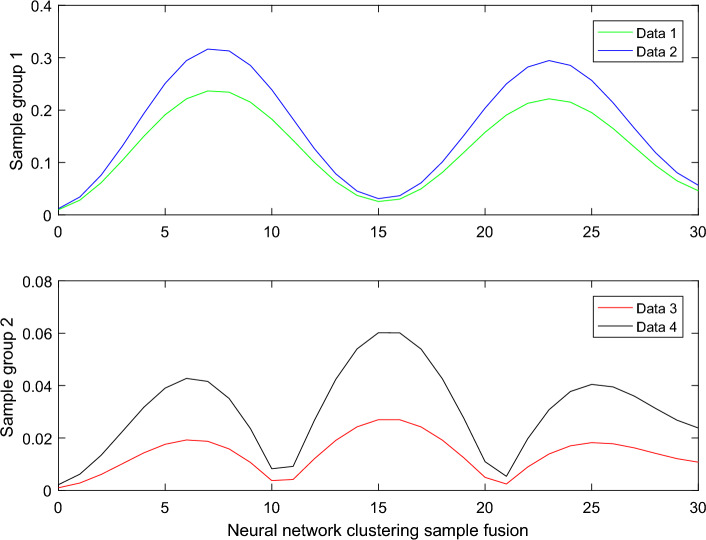
Neural network clustering sample fusion.

The algorithm first performs variance-based enhancement on color images, then uses the pulse-coupled neural network with spatial adjacency and similar brightness feature clustering, locates the noise points by comparing the difference between the ignition times of different image pixels, and finally follows the rules similar to the vector median filtering algorithm. Since each pixel will calculate the similarity with multiple seed points, the seed point that is most similar to the pixel point, that is, the corresponding minimum distance, is taken as the clustering center, and then the number of the seed point is given on the pixel point. Finally, the color value and coordinate value of the seed point and all pixel points are added and averaged to obtain the new cluster center in Fig. 1.

The registered right and left focus samples were fused. Effective fusion results should result in a clear left and right image, that is, restore the contrast and sharpness of the respective mode paste areas in the two images. In order to make it as consistent as possible with the physical standard graph, we choose the correlation coefficient between the perceptual result and the physical standard graph as one of the measurement indexes. In addition, the definition of the average gradient balanced image, the scale of the standard deviation balanced image and the information degree of the entropy balanced image are discussed. When the pulse coupling kernel slides to the entire input data, only local data is extracted each time for feature calculation, which reflects the local connectivity of PCNN and greatly speeds up the calculation speed. In the sliding process, the parameters of each pulse coupling core remain unchanged, which means that each pulse coupling core only observes the features it wants to obtain through its own parameters, which greatly reduces the number of parameters and reflects the parameter sharing property of PCNN.

### Homomorphic filtering analysis

Based on the chaotic sequence and cyclic/block diagonal splitting structure of homomorphic filtering, aiming at the problem of poor reconstruction performance and high computational complexity, this paper proposes a deterministic measurement matrix optimization strategy based on modified gradient descent to minimize the correlation between observation matrix and projection matrix. Then the point (x, y) belongs to the foreground, otherwise belongs to the background. Compared with single threshold segmentation miu(r, g, b), double threshold segmentation can effectively reduce misjudgment.

6 $$ miu(r,g,b) = \sqrt {(miu.\exp (r,g) - miu.\log (r,b)) - 1} $$

7 $$ \log (i + j) - \log (i - j) - 1 < i - j $$

Since the point cloud data log(i + j) has no clear connection relationship, the two-sided filtering algorithm can not be directly applied to the point cloud surface denoising. Bilateral filtering algorithm mainly involves point V. In this paper, the method is used to calculate the adjacent points of discrete point V, and the normal calculation of the vertex is obtained by optimizing a secondary energy term of the adjacent points.The essence of visual perception is that visual perception is divided into several regions according to some similarity principles, so the quality of segmented images can be judged by using the uniformity in each region. Therefore, the optimal segmentation result can be identified by calculating the 1/(1−i) value of the binary image, so as to realize the automatic selection of the optimal segmentation result exp(1/d).

8 $$ \frac{1 - i}{i}Z(i - j - k) = \frac{1}{1 - i} + \frac{1}{1 - j} + \frac{1}{1 - k} + 1 $$

9 $$ \exp ( - \frac{miu(x + y - 1)}{{2d}})/\exp ( - \frac{x + y}{d}) < 1 $$

Coupling connection miu(x + y-1)/d refers to the operation mechanism of PCNN when the connection strength coefficient is not equal to 0. In this case, the element not only receives external excitation, but also receives feedback input information of the neighborhood pulse element. In this case, each pulse element in the model is coupled to each other. In the case of coupling connection, using coupling connection input L to regulate feedback input F is the key to communication between pulse elements in the coupled PCNN model.

10 $$ \sum {|x + p(x - 1)|} \sum {|x - p(x - 1)|} \in w(x,t) $$

**Figure 2:**
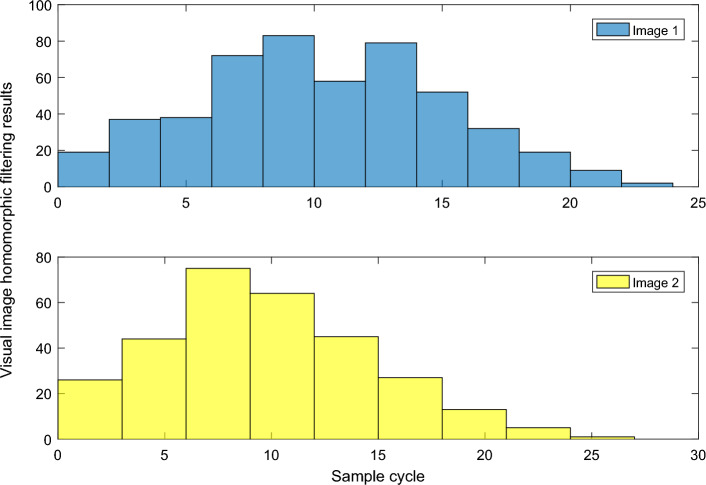
Homomorphic filtering results of visual images.

In the clipping method, the boundary p(x-1) of one grid is used to cut another grid in the overlapping area w(x, t), and then a new triangle is generated on the common boundary to make the two grids join together. This method will produce a large number of small triangles at the common boundary due to clipping. Moreover, this method only uses the vertices in one mesh in the overlapping region, and the vertices in the other mesh are completely abandoned. For the mesh with large overlapping region, the overlapping region of the two grids cannot be used to correct the vertices. At the same time, due to the error in the registration process of multi-slice grids, the boundary of one grid needs to be projected to another grid before clipping in Fig. 2.

Since the image fusion rules determine the final perception result, it is better to choose the appropriate fusion compliance rules that are more in line with the perception expectation to design the image perception experiment. We know that the image after pyramid decomposition will get the low frequency subgraph of near similar information of feature image and the high frequency subgraph of detail feature of feature image. Therefore, designing different perception rules for different features can better achieve high-quality image perception. For the same experimental image, if the entropy of the segmentation image obtained by a certain method is relatively large, it indicates that the performance of the segmentation method is better. In general, the segmentation effect of the proposed method is better than other segmentation methods. Whether it is objective evaluation criteria or direct observation of segmentation effect, it can be noted that the protection of color edge details in the center area is better than other methods.

### Pulse coupling calculation

Pulse coupling feed input is the main input source received by pulse elements, and neighboring pulse elements can influence the feed input signal of pulse elements through link mode. The external stimulus is received by the feed input domain and then coupled with the adjacent pulse element pulse signal received by the link input domain and sent to the internal activity item. The value of the internal activity term gradually increases with the cycle, while the dynamic threshold gradually decreases with the cycle t(i, j), and the value of the internal activity term is compared with the dynamic threshold for each cycle s(i ,j).

11 $$ A + B*t(i,j) + C*s(i,j) < 1 $$

12 $$ 10\log \;(2.5^{ \wedge } x - 2x - 1)^{ \wedge } 2 < 1/\log \;(2^{ \wedge } x - x) $$

In contrast log(2^x−x), as a simplified and improved model of PCNN model, LSCN (Long and Short Sequence Concerned Networks) continuously simplifies the input signal acquisition mechanism, and the total amount of undetermined parameters is greatly reduced. There are three leakage integrators in the traditional PCNN model, which need to perform two pulse coupling operations. In the LSCN model, there are also three leakage integrators, but only one pulse coupling operation is required. This determines that the time complexity of the LSCN model is lower than that of the traditional model, and it can be seen that the relationship between internal activity items and external incentives in this model is more direct. Not only that, different from traditional PCNN, the iteration process h(i, j)/x of LSCN model is automatically stopped rather than manually set, which is more convenient to operate in multiple iterations.

13 $$ \sqrt {\Delta h_{x} (i,j)/x + \Delta h_{y} (i,j)/y + \Delta h_{z} (i,j)/z} = 1 $$

14 $$ 1 - \ln \sum {|p(x) - p(x - 1)|} - \ln p(x) \in p(1 - x) $$

In the process of perception at this level p(x)−p(x−1), an independent preliminary judgment is made on each image and relevant conclusions are set up, and then each judgment and conclusion are perceived, so as to form the final joint judgment. The amount of data processed by the decision level perception method is the least among the three levels, and it has good fault tolerance and real-time performance, but it has more pre-processed data.

15 $$ X(a,b,c) = R(a,b)/c + G(c,b)/a + B(a,c)/b $$

Firstly, feature extraction X(a, b, c) is carried out on the original image, and then these features are perceived. Because the object perceived at this level is not the image but the characteristics of the image, it compreses the amount of data required to be processed to a certain extent, improves the efficiency and is conducive to real-time processing. The candidate regions, classification probabilities, and extracted features generated by the PCNN network are then used to train the cascade classifier. The training set at the initial time contains all positive samples and the same number of negative samples randomly sampled. The RealBoost classifier is followed by pedestrian classification.

**Figure 3:**
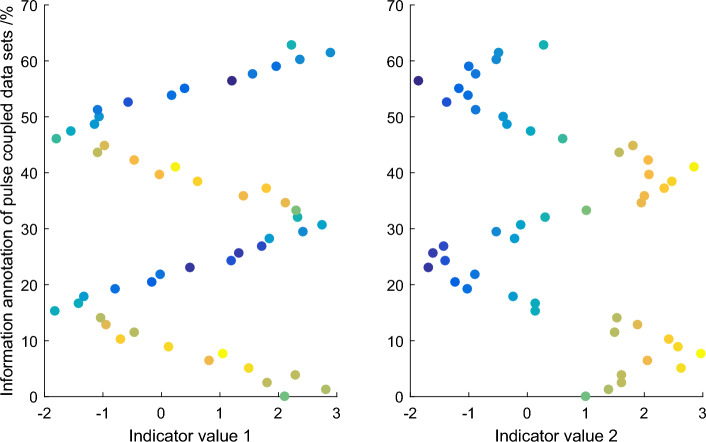
Information annotation of pulse coupling data set.

The audience dataset labels age and gender disaggregated information together, suggesting that the model is actually a multi-task model, but does not explore the intrinsic relationship between the two tasks for better detection results. The model in Fig. 3 had a gender identification accuracy of 66.8 percent on the audience dataset. However, these completely abandoned significance graphs actually contain some important significance information, which will cause the significance detection effect of PCNN model to be inaccurate. Therefore, it is necessary to reasonably perceive the significant information at each scale based on the significant information at the minimum entropy scale.Therefore, based on the saliency information at the minimum entropy scale, this paper takes the reciprocal of the corresponding entropy at other scales as the contribution rate to perceive the saliency information at other scales, so as to propose a multi-scale final saliency map determination method.

### Image enhancement dynamic processing

The visual boundary coefficient is more suitable for describing the difference between the visual boundary and the visual frame, and image enhancement is convenient for processing visual boundary detection. Based on the diffusion principle of nonlinear partial differential equation, the model can control the diffusion direction by introducing appropriate diffusion flux function, and can also be combined with other visual boundary detection methods. In order to verify that the superpixel-based unsupervised FCM color visual perception method proposed in this chapter can obtain the best segmentation effect, 50 images were selected from BSDS500 as experimental samples. Since the method proposed in this chapter can automatically obtain the cluster number C value, while the traditional clustering algorithm uses a fixed C value for each image, the fixed value of C and the method of automatically obtaining the cluster number C value will be used for the experiment respectively. The algorithm requires three essential parameters, namely, the weighting index, the minimum error threshold and the maximum number of iterations, which are respectively 2, 15 and 50 in this experiment, and the adjacent window size is set to 3*3.

**Figure 4:**
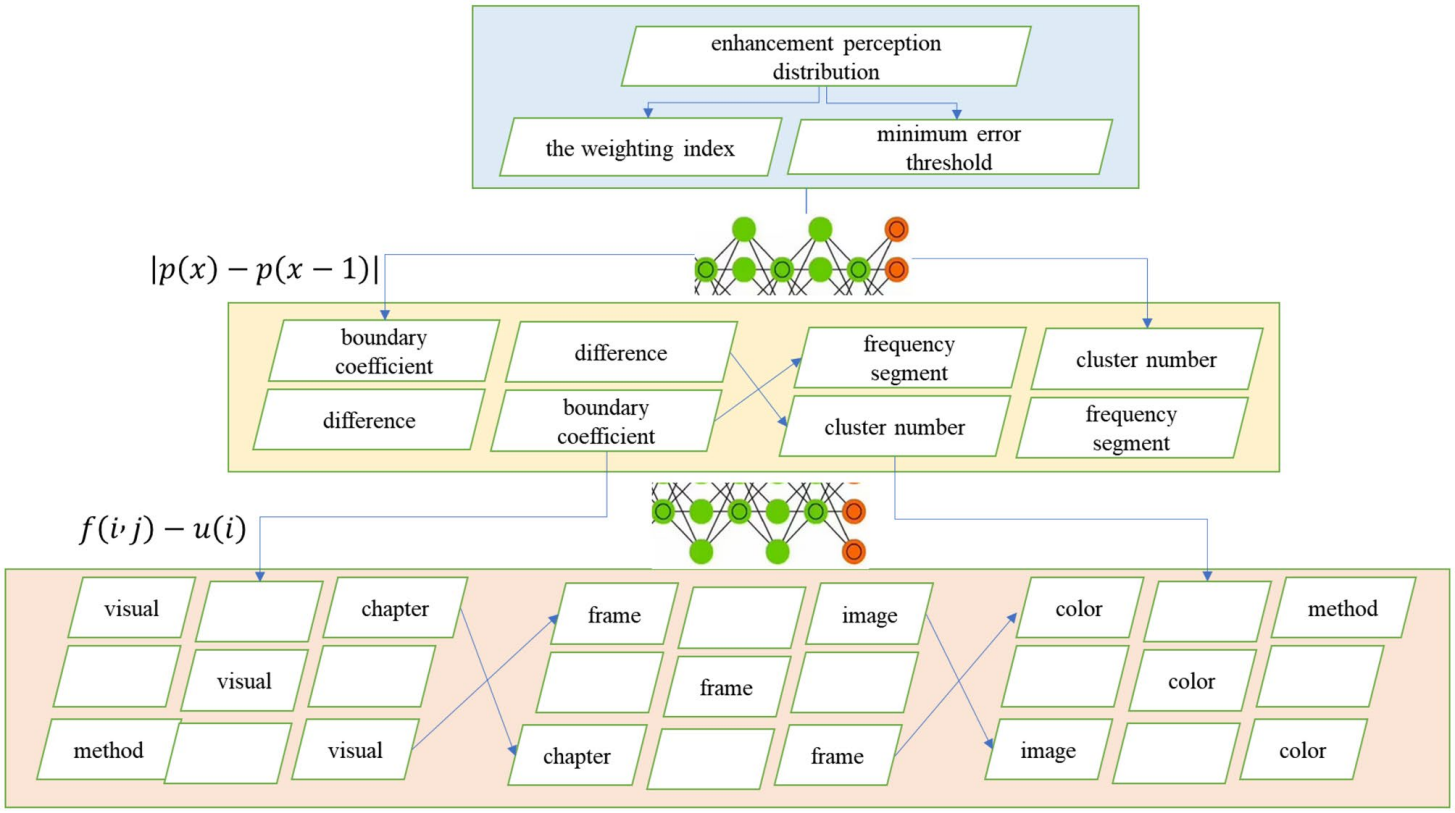
Image enhancement perception distribution.

As can be seen in Fig. 4, although the perceptual image obtained by the maximum value method is optimal in the optical brightness of the image, its edge has more obvious "sawtooth" phenomenon and is more blurred. Compared with the source image, the perception image obtained by the discrete wavelet transform method has obvious shortcomings in saturation and brightness. From the perspective of visual effect, the perceptual image obtained by the visual perception transformation method has obvious edge oscillation effect. In contrast, the proposed image perception algorithm based on compressed sensing theory has achieved good visual effects in terms of clarity, contrast and detail representation. Visual boundary detection method based on visual boundary coefficient has certain shortcomings in practical application, if the visual boundary neighborhood between frame and frame shear in irregular change, the visual border visual boundary coefficient decreases, and it is also possible for video clips in the visual dithering and make the visual boundary coefficient increases, this could reduce the detection performance of the algorithm.

If the minimum value of the interval in which the previous frame is located is equal to the minimum value of the minimum value of all subintervals in the search window, a further comparison is made in the subinterval in which the current frame is located. Since the search window of the current frame does not necessarily coincide exactly with the subinterval, the minimum value of the subinterval of the current frame boundary needs to be recalculated when determining the minimum value of the different subintervals (even without recalculation, the impact is limited).

### Visual perception tuning

Without the visual perception shared pulse coupling layer, P-Net's face detection and pedestrian detection will need to extract features from 224 × 224 pixel images respectively, and the time spent training these two tasks will be doubled, and R-Net with 448 × 448 pixel input will take even more time. At the same time, the internal connection of face detection and pedestrian detection has a special, most can locate face detection to the pedestrian detection box, so will face detection and pedestrian detection joint training can improve their accuracy. Obviously, it is simple and fast to segment PMA (Plane Moving Average) sequences according to 0 points, but many long motion patterns will be generated. Long motion mode is not conducive to key frame extraction, because it is difficult to express visual content according to long motion mode. Secondly, the long movement mode expressed by the triangular model will have a large error and is not accurate. At this point, we can separate the long motion mode into multiple motion modes. The method of separation is to determine the minimum point in the long motion pattern.

**Figure 5:**
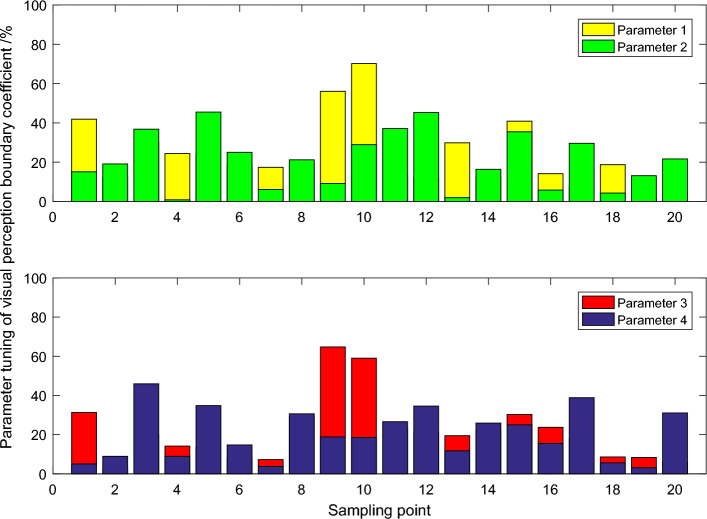
Parameter adjustment of boundary coefficient of visual perception.

It can be seen that the performance of visual boundary detection using visual boundary coefficient and standard histogram intersection method has its own advantages and disadvantages, and the overall performance is equivalent. For the data set in Fig. 5, the fixed min value detection method using visual boundary coefficients shows different properties. In the face of common noise attacks, the improved PCNN model achieves a higher Area Under Curve (AUC) value, which also indicates that the improved model has more robust robustness. If the cost of false visual boundary detection is equal to that of missed visual boundary detection, the visual boundary detection method using visual boundary coefficient is slightly inferior to the standard histogram intersection method on movie and video data sets. However, on the video dataset, the visual boundary detection method using visual boundary coefficients is slightly better than the standard histogram intersection method. If the cost of false and missed visual boundaries is not equal, the opposite is true. In general, the method using symmetric weighted window frame difference and moving average window frame difference is more stable and reliable than the method using 1/2- symmetric weighted window frame difference and 1/2- moving average window frame difference.

## Application and analysis of visual perception model based on pulse coupled neural network

### Pulse coupled neural network sampling

The pulse-coupled neural network is compared with the segmentation results of Kittler method and the traditional PCNN model to verify the effectiveness of the algorithm. Note that this paper has carried out a lot of testing work for this algorithm. Due to space limitation, only some typical image test results are listed. The operation principle of the next four pulse coupling layers is the same as the first layer, but the selected pulse coupling core size and sliding step size are different. Similarly, the pooling layer immediately after the second pulse coupling and the fifth pulse coupling has the same function and parameters as the pooling layer behind the first convolution layer. After the features are extracted by the 5-layer pulse coupling, F-Net uses two fully connected layers to integrate the extracted features. All pulse elements between the two layers of the fully connected layer have the right to reconnect, which is usually at the tail of the pulse coupling neural network to ensure that the features obtained through PCNN will not be lost.

**Figure 6:**
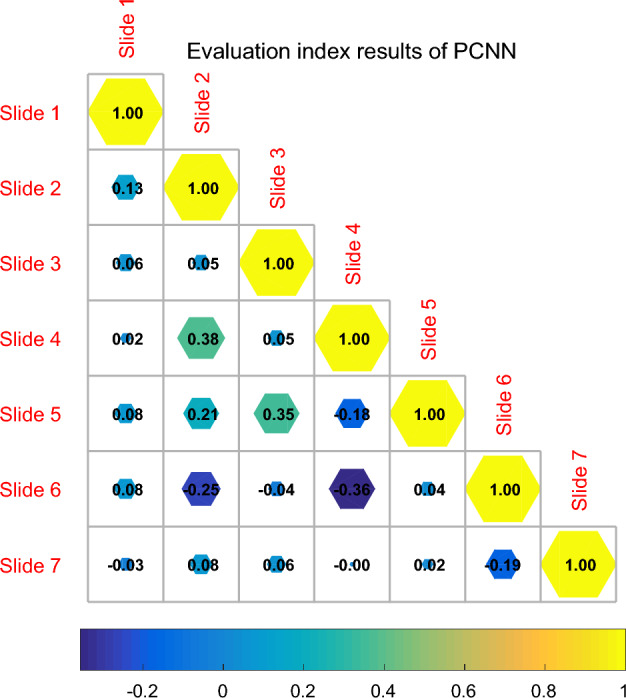
Evaluation index results of pulse-coupled neural network.

After obtaining the PMA sequence of Table 1, the next step is how to extract the movement pattern from the PMA sequence. The essence is to divide the PMA sequence into motion patterns. Since the motion mode consists of a motion acceleration process and a deceleration process, the PMA value at the starting and ending points of the ideal motion mode should be 0. For ease of processing, we use the triangle model to model the motion pattern, that is, the triangle model is used to segment the PMA sequence. In addition, it includes the product of feedback input and decay impairment at the last iteration and the product of output value and amplitude of adjacent pixels at the last iteration. The link input receives the product of the link input and the attenuation value and the product of the output value and the amplitude of the adjacent pixel at the last iteration. As can be seen from Fig. 6, the input of each time is related to the output of the last iteration and the adjacent impulse elements, which reflects the close connection of the model.

**Table 1:** Description of pulse-coupled neural network.

Sample nodes	The precision value	The recall rate	The error value
10	0.46044	0.51722	0.03662
20	0.62249	0.92411	0.33988
30	0.63896	0.65399	0.10451
40	0.85607	0.57696	0.38804
50	0.48609	0.14408	0.46745
60	0.75937	0.97943	0.06887
70	0.90884	0.93193	0.27186
80	0.44613	0.40358	0.11806

It can be seen that the results of objective evaluation indicators all show that the perception algorithm proposed in this paper is superior to other perception algorithms in retaining the details of the edge, line and contour of the source image. It can be seen that through the optimization of multi-perception strategy and measurement matrix, the new idea of multi-source image perception under the theoretical framework of PCNN can further improve the quality of image perception. This is because the method in this paper fully considers the correlation between pixels in visual perception, and adopts the idea of double threshold to further improve the segmentation ability of PCNN. Due to the interference of outdoor monitoring images and the complex image background environment, such as illumination changes and the existence of shadows, the segmentation of such images is a difficulty in the field of visual perception. It can be seen that Kittler algorithm is almost ineffective for the segmentation of such images. Meanwhile, the comparison figure shows that the method in this paper can clearly segment the pedestrian area, and the pedestrian contour is complete, the action posture is clear, and the object with a large number of details in the background also has a good segmentation effect. It can be seen that the PCNN algorithm with double threshold greatly improves the segmentation performance of the traditional PCNN algorithm, and can better reflect the shape features of the target.

### Simulation of visual perception model

In this paper, Matlab R2010a is used as the simulation environment, and the outdoor monitoring capture image is used as the representative to test the algorithm. The quality of iteration effect has a great relationship with the number of iterations. If the number of iterations is too large, computing resources will be wasted, and if the number of iterations is too small, the algorithm will not run adequately. Although Selective Search strategy is faster and better than brute force search and segmentation algorithm in candidate region extraction, a lot of time is still spent on candidate region extraction in the target detection process using Selective Search strategy. For example, it takes 2 s to extract candidate regions for each image by using Selective Search strategy in Fast R-PCNN model, but only 0.2 s to extract and classify all candidate regions by using pulse coupled neural network later.

**Figure 7:**
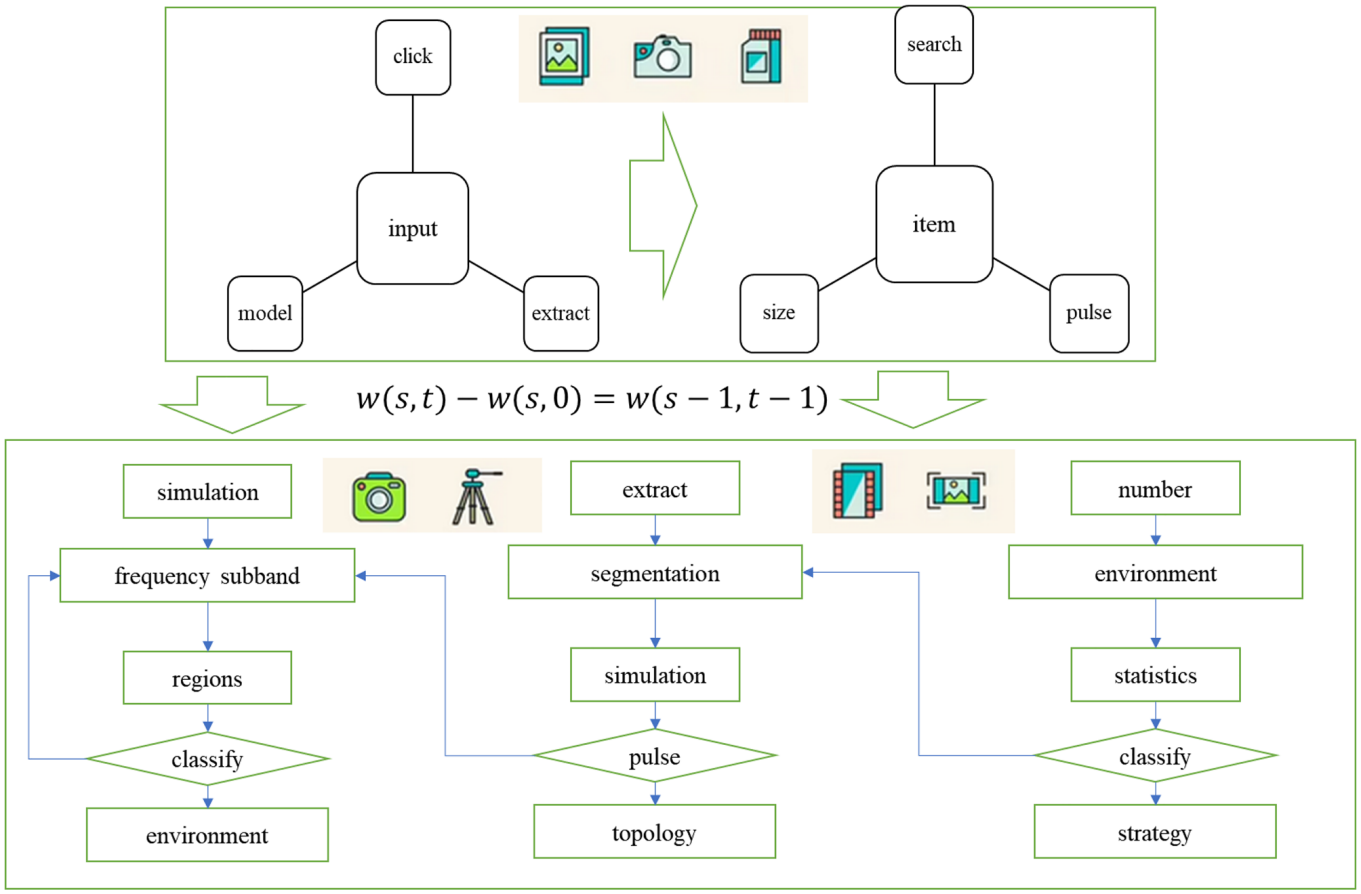
Visual perception network topology.

The difference between the LSCN algorithm using ignition frequency as output and the algorithm using L item as output in Fig. 7 is only in the output item, so as to compare the results of the LSCN model using ignition frequency and L item to guide perception respectively. The traditional algorithm selects the pixel value of the perceptual image through the size of the ignition frequency rate.

The module has 15 display boxes, which respectively display the high and low frequency subband images of image A, B after NSST (Non-downsampled Shear Wave Transform) decomposition, the high and low frequency subband images after fusion, and the final perception results. Click button 1 to input the image to be sensed, click button 2 to perform NSST decomposition on images A and B to obtain the corresponding high and low frequency subgraphs, and click button 3 to perceive the corresponding high and low frequency of images A and B respectively. The algorithm in this chapter and the LSCN algorithm of frequency division use the size of L item to determine the pixel value of the perceptual image. The algorithm in this chapter is compared with four traditional algorithms, the purpose is to compare the effect of using the improved LSCN model and the traditional perception algorithm.

### Case application and analysis

The example first initializes the shared pulse coupling layer using the model pre-trained on ImageNet, and then sets the input data path to the Wider Face training data set, and sets 1 picture as a batch. The images in each batch extract shared features through the shared pulse coupling layer, and focus on extracting face features through the special pulse coupling layer for face detection. After feedforward calculation to the last layer of F-RPN, the face classification loss and border regression loss are calculated, and then the loss is transmitted back and the parameters of the shared convolution layer and F-RPN are updated.

Here, the number of training data sets is set to 4 times, so the training is continued until the 4 training of all images is completed. All points in the data set are marked on the graph, and the graph formed by these points is called the decision graph. In Table 2, the points with large values, above the coordinate plot and far from the bottom dense area are selected as the clustering center. However, when there is a series of continuous sparse points in the decision graph, it is very difficult to select the appropriate clustering center.

**Table 2:** Impulse coupled neural network decision.

Network mode	The front-end web	In the terminal network	Between the terminal network	Back-end network
PCNN module 1	1*3 conv	1*3 concat	3*3 conv	3*3 couple
PCNN module 2	3*3 conv	3*3 concat	6*3 conv	6*3 couple
PCNN module 3	6*3 conv	6*3 concat	9*3 conv	9*3 couple
PCNN module 4	6*6 conv	6*6 concat	6*6 conv	6*6 couple
PCNN module 5	6*9 conv	6*9 concat	9*6 conv	9*6 couple

AT is defined as the highest target segmentation accuracy that can be achieved when using superpixels as units, and the proportion of labeled pixels that do not leak from the groundtruth boundary is calculated by labeling each superpixel with the groundtruth fragment with the largest overlap surface product. Its range is [0,1], and the larger the value, the better, indicating that the superpixel overlaps more with the object in the image. Then when the sliding window slides on the image, the center point of the current sliding window can be recorded every time it reaches a new position.

With the position rules of candidate regions relative to the sliding window and the central point position of the sliding window, the position of all candidate regions can be reproduced at any time. The Gabor filtering texture extraction method and PCNN time signature extraction method selected in this paper have certain biological theoretical basis, which makes the features of the extracted salient region more biological credibility. Finally, the multi-classification method of support vector machine is used to carry out experiments on the related image library. The classification results show that the introduction of visual saliency into image classification can reduce the complexity of calculation, and the classification accuracy is 94.26%, which is 4.3% higher than the classification of original image features.

**Figure 8:**
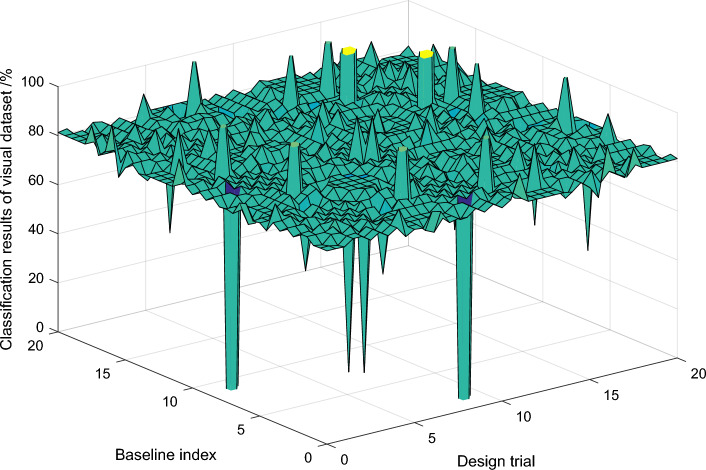
Classification results of visual perception training data set.

Due to the high definition of the pictures used in the task data set and less change of human posture, both networks achieve good detection accuracy, and the optimized PCNN is slightly better than the serial network. However, in terms of single detection time, the optimized PCNN takes nearly one third less detection time than the series network. Therefore, the PCNN in Fig. 8 can complete the detection of multiple tasks at the same time in a short time, and maintain or even improve the detection rate. The AT value of the method in this paper is larger than that of other methods in the three measured images, reaching 0.9544 and 0.9832 respectively, indicating that the integrity of boundary retention is good, the contour of target object can be better extracted, and the segmentation result is accurate and reliable. It can be seen from the average time comparison of each algorithm when the segmentation number is 300 in 50 images in the image library that the segmentation time of the algorithm in this paper is relatively long, mainly because the introduction of iterative calculation of the internal active item U value of neighborhood pixels consumes more time. However, according to the comparison results of each index and the segmentation effect, the segmentation method proposed in this paper has certain advantages over other segmentation methods. By comparing with a variety of algorithms, it is concluded that the algorithm in this paper is better than the comparison algorithm in terms of both human eyes and objective evaluation indicators.

## Conclusion

In view of the traditional figure like segmentation algorithm under complex background environment division, such as low precision and poor anti-interference problems, this paper constructs the visual perception model based on pulse coupled neural network, using PCNN model coupling characteristic of pulse point, considering the correlation between the pixels, introduced the threshold ideas for improved PCNN model. Firstly, the motion field of the image frame is estimated in the time domain, and then the global motion region and local motion region are obtained by the global motion estimation method based on the six-parameter affine motion model, which are used for visual perception operation type query or global motion query. When the traditional model is applied to image segmentation, the effect is not ideal if only relying on the synchronous oscillation characteristics of the model. The visual perception model of pulse coupled neural network (PCNN) is found and proposed in the study of the pulse synchronous oscillation phenomenon of mammalian visual cortex neurons. The characteristics of the model are consistent with the current progress of biological vision research, but the model has shortcomings in computational efficiency. This limits the application of the model to other technical domains. So without changing the excellent characteristics of the model, how to simplify the model and improve its algorithm is the focus of the next step.The local motion region is then segmented by multi-stage affine motion consistency to obtain independent motion region. In the spatial domain, the system is mainly divided into different reconstruction methods of compressed sensing, non-downsampled shear wave transforms, image perception and other modules. At the same time, the region uniformity measure function is introduced to realize the automatic selection of the optimal segmentation results. Experimental results show that compared with the traditional model, the segmentation effect, anti-interference, operation speed and stability of the visual perception model based on pulse coupling neural network proposed in this paper have been improved to varying degrees.

## Data Availability

The data used to support the findings of this study are included within the article.
